# Supplementary material for: Identification of Cyclin L1 as a Host Factor Regulating Hepatitis B Virus Replication
Source: Viruses. 2026 May 8;18(5):545. doi: 10.3390/v18050545 (PMC13211425; doi:10.3390/v18050545)
Supplement: Supplementary file 1 [file viruses-18-00545-s001.zip › viruses-4262821-supplementary.pdf]

Supplementary materials

Manuscript with highlighted changes

Identification of Cyclin L1 as a host factor regulating Hepatitis B Virus replication

Collins Oduor Owino[1,2,3], Balakrishnan Chakrapani Narmada[1,4], Gian Yi Lin[4], Pauline Poh Kim Aw[1], Nivrithi Ganesh[1], Jovi Tan Siying[1], Marie-Laure Plissonnier[5], Thangavelu Thangavelu Matan[4], Niranjan Shirgaonkar[1], Juan Pablo Bifani[2,6,7], Massimo Levrero[5], Giridharan Periyasamy[3], Seng Gee Lim[8,9,10], Ramanuj DasGupta[1*]

* Correspondence to R.D.

Affiliations

1 Laboratory of Precision Medicine and Cancer Evolution, Genome Institute of Singapore, Agency for Science, Technology, and Research (A*STAR), 60 Biopolis St., #02-01 Genome, Singapore 138672

2 Infectious Diseases Translational Research Programme, Department of Microbiology and Immunology, Yong Loo Lin School of Medicine, National University of Singapore, Singapore

3 Department of Biochemistry, **Faculty** of Science and Technology, **University of Nairobi**. P.O.BOX 30197-00100, **Nairobi**, Kenya

4 Experimental Drug Development Centre, A*STAR, 10 Biopolis Way, Chromos, Singapore 138670, Singapore

5 Cancer Research Center of Lyon, INSERM U1052, Lyon University, Hospices de Lyon, Lyon, France

6 A*STAR Infectious Diseases Labs, Agency for Science, Technology and Research (A*STAR), Singapore, Singapore

7 Lee Kong Chian School of Medicine, Nanyang Technological University, Singapore, Singapore

8 Department of Medicine, Yong Loo Lin School of Medicine, National University of Singapore, Singapore

9 Institute of Molecular and Cell Biology, A*STAR, 61 Biopolis Drive, Proteos, Singapore, 138673, Singapore

10 Division of Gastroenterology and Hepatology, National University Hospital, National University Health System, Singapore

Fig. S1 Analysis of HBV mediated transcriptomic changes

A. Down-regulated pathways upon HBV infection in PHH at 96hrs compared to the mock control. RNA or protien was extracted from liver tissue from CHB patients and patient who have achieved surface antigen loss (HBsAg negative) and HBV pgRNA quantified by RT-qPCR and the expression normalized to beta actin. **D.** Protein was extracted from the liver tissues and 30μg of RNA analyzed in Western Blot to show the levels of *CCNL1* in HBsAg negative and CHB samples. **B′.** Quantification of band intensity for *CCNL1* normalized to the beta actin levels for the CHB and HBsAg negative patients. **C.** Levels of *CCNL1* mRNA in HBsAg negative, HBeAg- and HBeAg+ samples quantified by RT-qPCR and normalized to the levels of beta actin.

Fig. S2 Evaluating the role of *CCNL1* during HBV infection

A. Quantification of the CCNL1 band intensity and normalized to GAPDH housekeeping gene. **B.** siRNA knockdown of Cyclin L1 did not show toxicity in HepAD38.7 cells. **C.** Representative Western Blot image showing the levels of the HBV core protein. **D.** Validation of the *CCNL1* knockdown by shRNA. **D′.** Quantification of CCNL1 band intensity and normalized to the GAPDH levels and expressed as a percentage to the control cells. **E**. IFA staining of CCNL1 in HBV-infected cells compared to control cells. **E′**. Quantification of the CCNL1 expression intensity in infected vs control cells. Statistical significance shown as p-value was analyzed by student t- test in GraphPad prism 8. Data are represented as mean ± SEM (n=3), ****p<0.0001, ***p<0.001, ** p<0.01, *p<0.05, ns; non-significant.

Fig. S3 Functional validation of Cyclin L1 in PHH

A. Validation of Cyclin L1 knockdown in PHH by Western Blot. **B.** Levels of *CCNL1* at mRNA level by RT-qPCR. **C.** Bright field images of sh-*CCNL1* and sh-control transfected PHH at day 9 (6dpi) and 10 (7dpi) in culture. The scale bar is 50μm. Statistical significance shown as p-value was analyzed by student t- test in GraphPad prism 8. Data are represented as mean ± SEM (n=3), ****p<0.0001, ***p<0.001, ** p<0.01, *p<0.05, ns; non-significant.

Fig. S4 Ectopic expression of *CCNL1* in HepG2-NTCP cells enhances HBV gene expression

A. Cell viability for the control and cells overexpressing Cyclin L1. **B.** Expression of *CCNL1* at mRNA levels as quantified by RT-qPCR. C. Validation of ectopic expression of CCNL1 by Western Blot. **C′.** Quantified CCNL1 band intensity for the control and overexpression cells and expressed as a percentage to the control. **D.** Levels of HBV pgRNA in control and HepG2-NTCP cells overexpressing *CCNL1* are shown. **E.** Levels of HBe antigen quantified by CLIA are shown. **F**. HBV extracellular DNA levels are shown as quantified by qPCR. Statistical significance shown as p-value was analyzed by student t- test for two groups and one-way ANOVA for more than two groups in GraphPad prism 8. Data are represented as mean ± SEM (n=3), ****p<0.0001, ***p<0.001, ** p<0.01, *p<0.05, ns; non-significant.

Fig. S5: Evaluating the role of Cyclin L1 on HBV RNA splicing

A-C. Levels of HBV spliced RNA variants in HepG2-NTCP and HepAD38.7 in both knockdown and overexpression models. **D.** Agarose gel displaying products of RT-qPCR of HBV Sp1 in the three categories of patients.

Fig. S6: Cyclin L1 interacts with HBV RNA

A. The HBV singly spliced RNA (Sp1) levels in the knockdown (shCCNL1) cells were expressed as a percentage of Sp1 levels in sh-control cells. **A′.** The splice ratio analysis wherein the levels of Sp1 in control and knockdown cells was calculated by dividing the expression of the Sp1 with the total HBV RNA+ Sp1 for the respective model in PHH. **B**. Nascent HBV RNA in HepAD38.7 cells. **C**. Increased phosphorylation of RNAPII in CCNL1 OE expressing cells. D. ChIP pulldown of phosphorylated RNA polymerase 5 in control and CCNL1 knockdown cells. **E and F** Pulldown of Histone H3ac (39139) and Histone H3ac in both control and CCNL1 knockdown cells. **G and H.** RNA immunoprecipitation (RIP) using anti-*CCNL1* antibody and IgG control were used for pulldown and the levels of S, X and core RNA quantified by RT-qPCR from Huh7-transfected and HepG2-NTCP models respectively. Data are represented as mean ± SEM (n=3), ****p<0.0001, ***p<0.001, ** p<0.01, *p<0.05, ns; non-significant.

Fig. S7 Morphometric analysis of HepG2-NTCP cells after Cell Painting and hierarchical clustering display two different clusters of sh-*CCNL1* and sh-control cells

PCA showing distinct clusters of sh-control and sh-*CCNL1* knockdown cells from cell painting analysis in HepG2-NTCP infected with HBV.

Table S1: Patient details

Patient ID	HBsAg	TX	HBeAg	DNA
Patient-1	non s-loss	Naïve	neg	10200
Patient-2	non s-loss	Naïve	neg	1342
Patient-3	non s-loss	Naïve	neg	21023
Patient-4	non s-loss	Naïve	Pos	8.95 log
Patient-5	non s-loss	Naïve	pos	3130000
Patient-6	non s-loss	Naïve	pos	19500000
Patient-7	s-loss	ADV>LAM>IFN>ETV>OFF Therapy	Neg	0
Patient-8	s-loss	Add On arm	Neg	0
Patient-9	s-loss	ADV > ADV/IFN >NIL	Neg	0
Patient-10	s-loss	ETV > IFN	Neg	0

Methods: Supplementary

Nascent RNA Assay

Actinomycin D (Invitrogen, A7592) was added to the culture media of the wells at a final concentration of 0,79 µM (1/1000, photosensitive) and incubated for 1-hour. After 1 hour at 37°C, 5-bromouridine (BU, SIGMA 850187) was added to culture media at a final concentration of 2 mM for 2h at 37°C. After the 2-hours incubation, the media was aspirated, and the cells washed 1X with PBS and thereafter lysed the cells with 300 µl of RA1 + DTT. RNA was extracted according to Qiagen RNA extraction kit protocol. In the meantime, 25 µl of Dynabeads goat anti-mouse IgG (Life Technologies, 1033) per sample was washed with PBS-BSA 0.1% supplemented with SUPERaseIn (Invitrogen, AM2696). After the washing, 1 µg of anti-BrdU antibody (BD Pharmingen 555627) was added and further incubated for 1 hour at room temperature on a rotary agitator. 1 µg of total RNAs was incubated for 10 minutes at 80°C and immediately put tubes on ice thereafter. The total RNAs were precleared with 5 µl of Dynabeads in a final volume of 400 µl of PBS-BSA 0.1% + SUPERaseIn and incubate 1 hour at 4°C on a rotary agitator. The beads were collected on a magnet and the supernatant incubated with Dynabeads conjugated with BrdU antibody overnight at 4°C on a rotary agitator. After overnight incubation, the beads were washed 3X 5 minutes with PBS-BSA 0.1% + SUPERaseIn. The nascent RNAs were eluted by adding 100 µl of water + SUPERaseIn and incubated for 10 min at 95°C. The nascent RNAs were purified and concentrated following the RNA clean and concentrator kit (Zymo Research, ZR1017) instructions and the RNAs eluted in 25 µl final volume. The RNAs were

reverse transcribed using Superscript VILO cDNA synthesis kit (Cat# 11754050, Invitrogen) and qPCR performed as described earlier using SYBR, pgRNA primers and RPLPO as a control.

Cell Painting Protocol

To evaluate the effect of *CCNL1* knockdown on HepG2-NTCP cell morphology, we carried out a cell painting assay as described earlier [44]. Step by step protocol is described below

1. Seed cells into 384-well plate in growth media (50 μL cell suspension/well) at a density of 600 cells/ well
2. Incubate overnight at 37°C, 5 % CO_2 to allow overnight recovery and growth of plated cells
3. Incubate the plates at 37°C, 5% CO_2 for 48 hours
4. Prepare MitoTracker staining solution from 1 mM stock:
    a. Add 3 μL of 1 mM stock per 1 mL of media
5. Do NOT remove media from wells! Add 10 μL of diluted MitoTracker staining solution and spin at 500g for 1 minute
6. Incubate in the dark for 30 minutes at 37C, 5% CO_2 (in incubator)
7. Add 20 μL of 16% PFA to achieve final concentration of 4% PFA and spin at 500g for 1 minute
8. Incubate for 20 minutes at RT (in drawer)
9. Remove supernatant and wash 2x with 1X HBSS, 70 μL/well (
10. Prepare permeabilization & staining solution (1x 384-well plate: 10ml):
    a. 0.1% Triton X-100, 1% BSA (w/v) in 1X HBSS (10 mL)
        - Since BSA may contain contaminants, prepare filtered 3% BSA in HBSS solution
    b. Phalloidin (12.5 μL)
    c. Concanavalin A (10 μL)
    d. Hoechst (1 μL)
    e. WGA (15 μL)
    f. SYTO14 (12 μL)
11. Add 20 μL of permeabilization & staining solution, incubate for 30 min at RT
12. Discard solution and wash 2x with 1X HBSS, 70 μL/well
13. Fill the wells with 60 μL of 1X HBSS or 0.05% Sodium azide (for long term storage)
14. Seal plates tightly with adhesive seals

Analysis of HBV spliced RNA variants

The fifteen known HBV splice variants were analyzed as described recently by Chabrolles and colleagues [41]. Quantitative qPCR was performed to detect HBV variant RNA including the unspliced RNA and the data normalized to the GAPDH and beta-actin genes in both knockdown and ectopic expression of *CCNL1*. The primers that detect all 15 spliced RNA (sv1 to sv15) and intron1, 2, and 2b were designed by Sommer and colleagues [50]. To further analyze splicing events upon knockdown or ectopic expression of *CCNL1*, the singly spliced HBV RNA (sp1) primers (SP1, 5′-TGCCCCTATCTTATCAACAC-3′ (nt 2311–2330, sense) and SP2, 5′-CAAATGGCACTAGTAAAC-3′ (nt 695–678, antisense)) were used to assess spicing as described earlier by

Sommer and colleagues [50]. These primers were also used to assess the levels of HBV spliced variant RNA in patient samples.

RNA Immunoprecipitation

In brief, 10X106 Huh7 were seeded into 15cm dish, and after overnight incubation, they were transfected with tet-wildtype HBV or tet-DPRE HBV plasmid. Media changed 16 hours post-transfection, and the cell lysates were collected 48 hours later. Alternatively, 10X106 NTCP-HEPG2 cells were seeded in 15cm dish and cultured for 96 hours and infected the following day with HBV at MOI of 3000 and further incubated for 4 days with media change every day. The cells were lysed in RNA lysis buffer and frozen at -80°C. Next, 5 µg of *CCNL1* or rabbit IgG antibody were added to pre-washed magnetic beads and incubated at room temperature, rotating for one hour. This was followed by washing of unbound antibodies twice with RIP wash buffer. DynaMag-2-magnet separator (Life technologies) was used during each wash to collect the beads and to allow the discarding of the supernatant. Finally, the supernatants were mixed with beads-antibody complexes or magnetic beads without antibody and incubated overnight, rotating at 4°C. The beads were washed 6 times with RIP wash buffer, and 50 µL of the beads suspension was aliquoted to test the immunoprecipitation efficiency by Western blotting. This was followed with proteinase K digestion and RNA extraction following phenol: isoamyl, chloroform method and precipitated with absolute Ethanol. 1µg of RNA precipitated RNA, and the 10% input were reverse transcribed using SuperScript III Reverse Transcriptase (Invitrogen, Carlsbad, CA, USA). Primers for HBV pgRNA; HBV pgRNA F: CGTTTTTGCCTTCTGACTTCTTTC, HBV pgRNA R: ACAGAGCTGAGGCGGTGTCTA were described earlier[16]. The following primers described previously by Sekiba and colleagues were used to detect the S, X, and core RNAs; S, Fwd, 5′-ACCTCTATGTATCCCTCCTG-3′ and Rev, 5′-GACTCAAGATGCTGTACAGAC-3′; X, Fwd, 5′-ACTCTCTCGTCCCCTTCTCCG-3′ and Rev, 5′-AGGCAGAGGTGAAAAAGTTGC-3′; and core, Fwd, 5′-ACAGTTATAGAGTATTTGGTG-3′ and Rev, 5′-GAGATCTTCTGCGACGCGGCG-3′[63].

References

44. Bray, M.A.; Singh, S.; Han, H.; Davis, C.T.; Borgeson, B.; Hartland, C.; Kost-Alimova, M.; Gustafsdottir, S.M.; Gibson, C.C.; Carpenter, A.E. Cell Painting, a High-Content Image-Based Assay for Morphological Profiling Using Multiplexed Fluorescent Dyes. *Nat. Protoc.* **2016**, *11*, 1757–1774, doi:10.1038/nprot.2016.105.

41. Chabrolles, H.; Auclair, H.; Vegna, S.; Lahlali, T.; Pons, C.; Michelet, M.; Couté, Y.; Belmudes, L.; Chadeuf, G.; Kim, Y.; et al. Hepatitis B Virus Core Protein Nuclear Interactome Identifies SRSF10 as a Host RNA-Binding Protein Restricting HBV RNA Production. *PLoS Pathog.* **2020**, *16*, e1008593, doi:10.1371/journal.ppat.1008593.

50. Sommer, G.; Heise, T. Posttranscriptional Control of HBV Gene Expression. *Frontiers in Bioscience* 2008, *13*, 5533–5547.

16. Wang, H.; Liu, K.H.; Fang, B.A.M.; Wu, H.Q.; Li, F. Di; Xiang, X.G.; Tang, W.L.; Zhao, G. De; Lin, L.Y.; Bao, S.; et al. Identification of Acetyltransferase Genes (HAT1 and KAT8) Regulating HBV Replication by RNAi Screening. *Cell Biosci.* **2015**, *5*, 66, doi:10.1186/s13578-015-0059-1.

63. Sekiba, K.; Otsuka, M.; Ohno, M.; Yamagami, M.; Kishikawa, T.; Suzuki, T.; Ishibashi, R.; Seimiya, T.; Tanaka, E.; Koike, K. Inhibition of HBV Transcription From CccDNA With Nitazoxanide by Targeting the HBx–DDB1 Interaction. *CMGH* **2019**, *7*, 297–312, doi:10.1016/j.jcmgh.2018.10.010.
