# Supplementary material for: Identification of Cyclin L1 as a Host Factor Regulating Hepatitis B Virus Replication
Source: Viruses. 2026 May 8;18(5):545. doi: 10.3390/v18050545 (PMC13211425; doi:10.3390/v18050545)

Fig. S1 Analysis of HBV mediated transcriptomic changes and CCNL1 expression CHB and infected cells

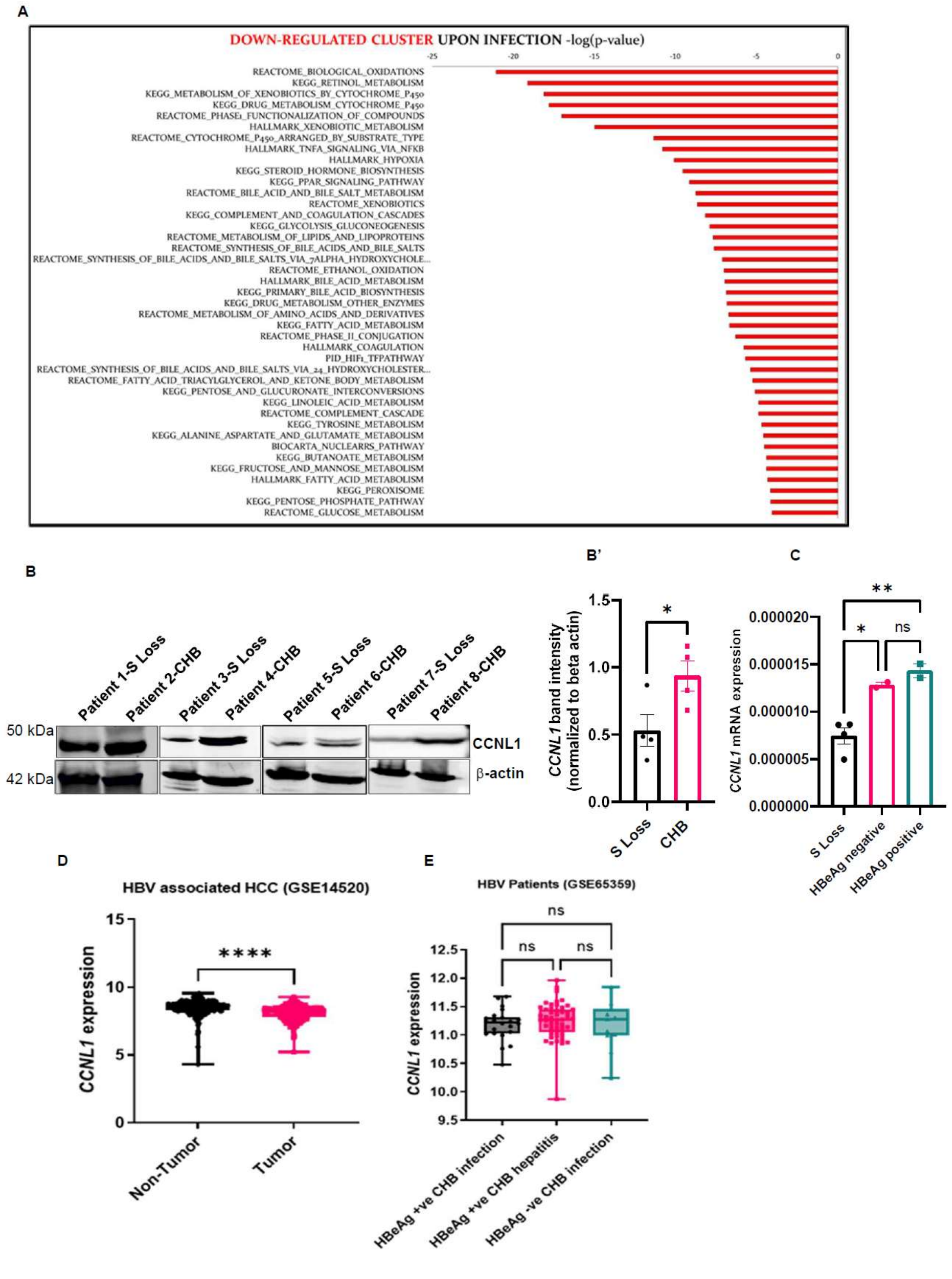

Fig. S2 Evaluating the role of *CCNL1* during HBV infection

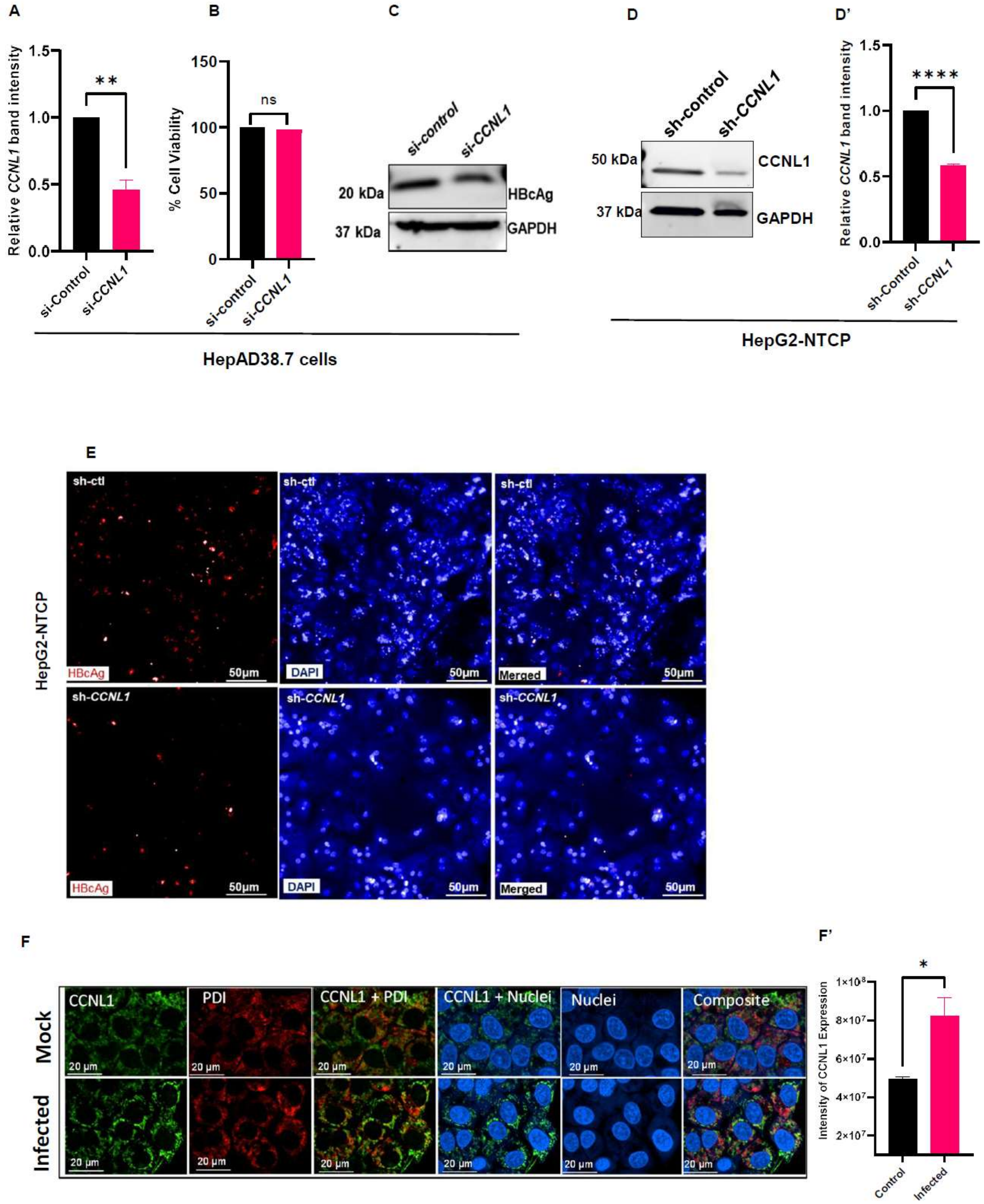

Fig. S3 Functional validation of Cyclin L1 in PHH

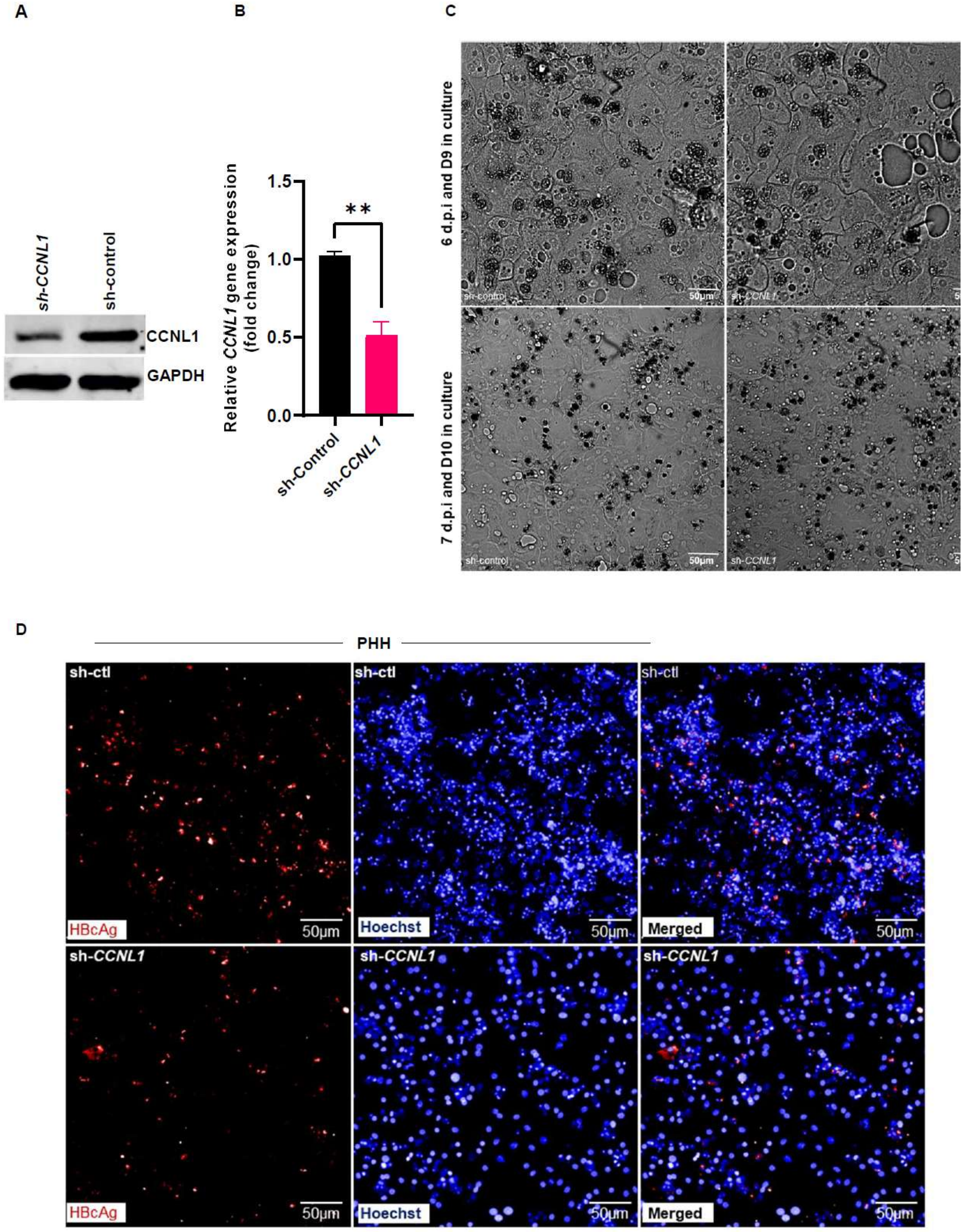

Fig. S4 Ectopic expression of *CCNL1* in HepG2-NTCP cells enhances HBV gene expression

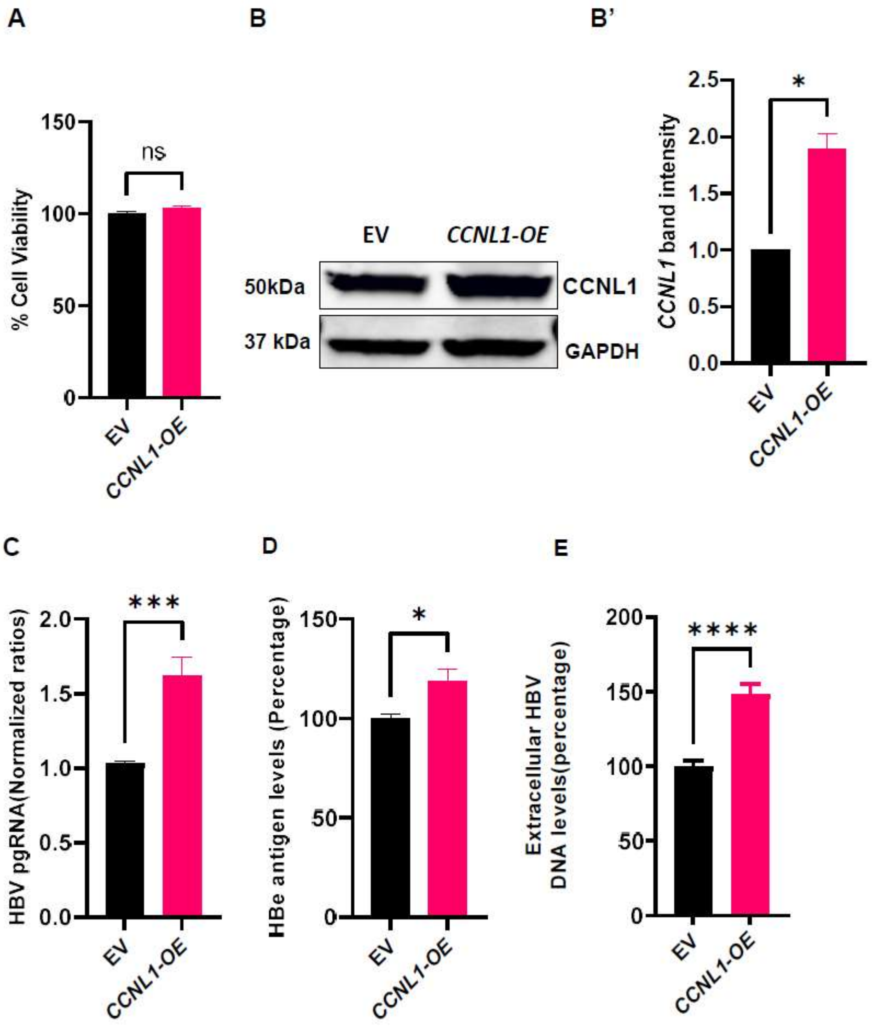

Fig. S5: Evaluating the role of Cyclin L1 on HBV RNA splicing

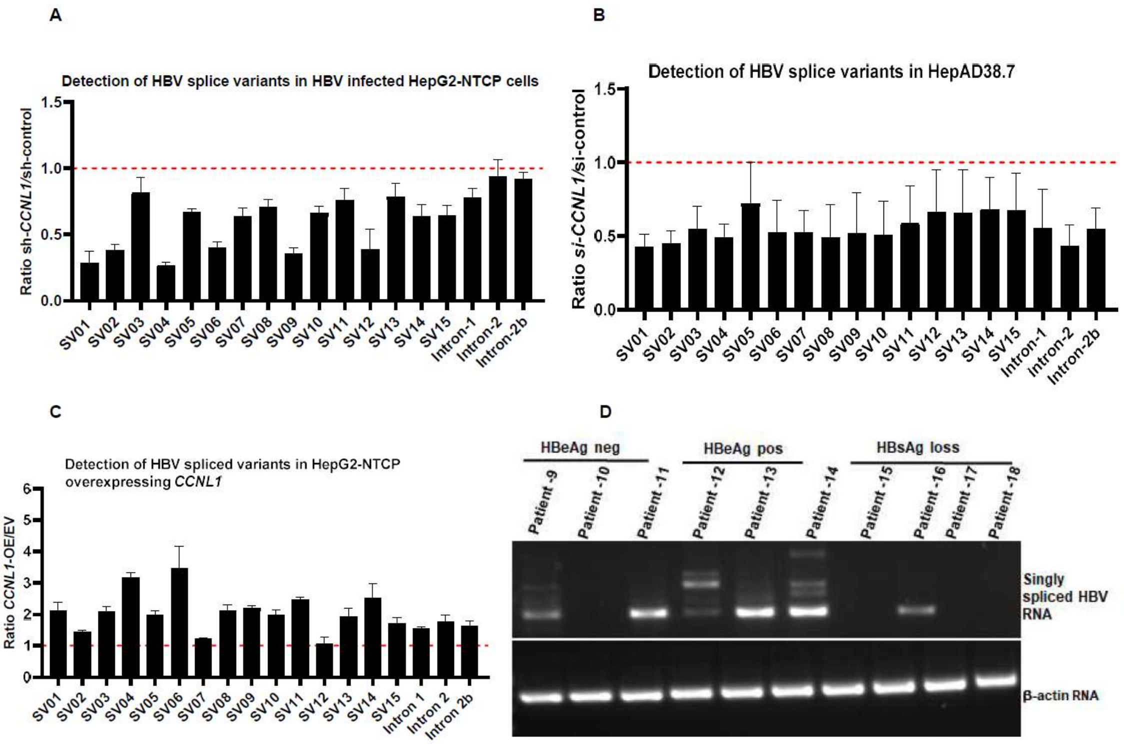

Fig. S6 Cyclin L1 regulates HBV transcription

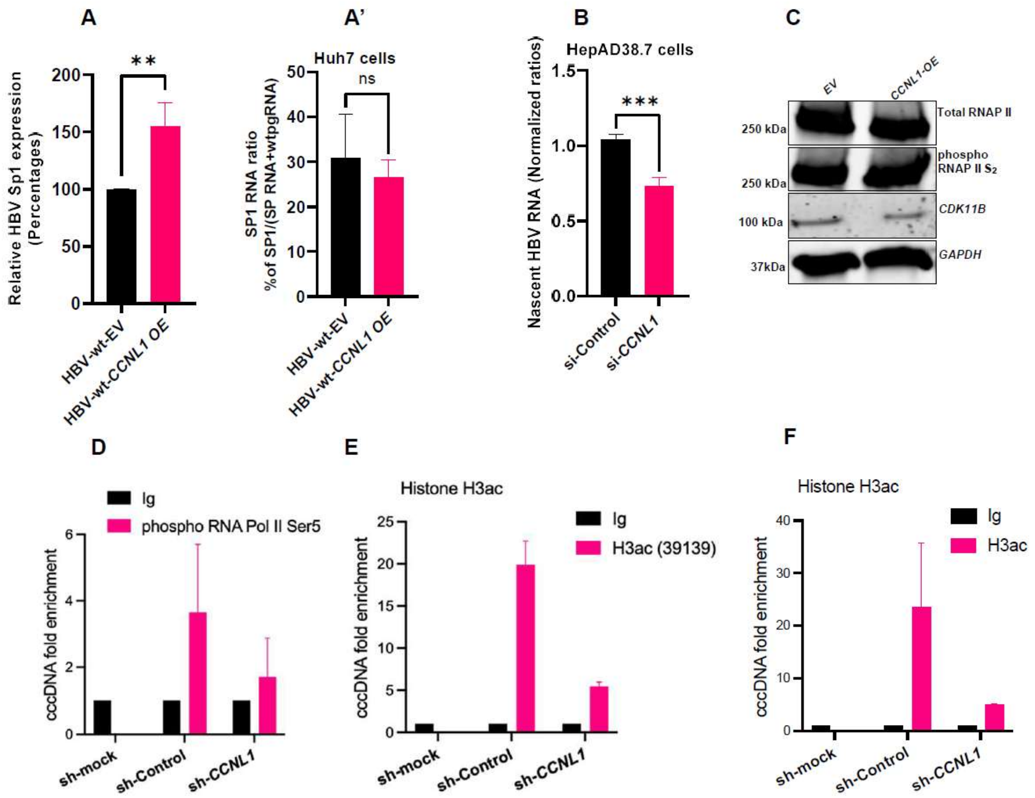

Fig. S7 Morphometric analysis of HepG2-NTCP cells after Cell Painting and hierarchical clustering display t[illegible] clusters of sh-*CCNL1* and sh-control cells

A

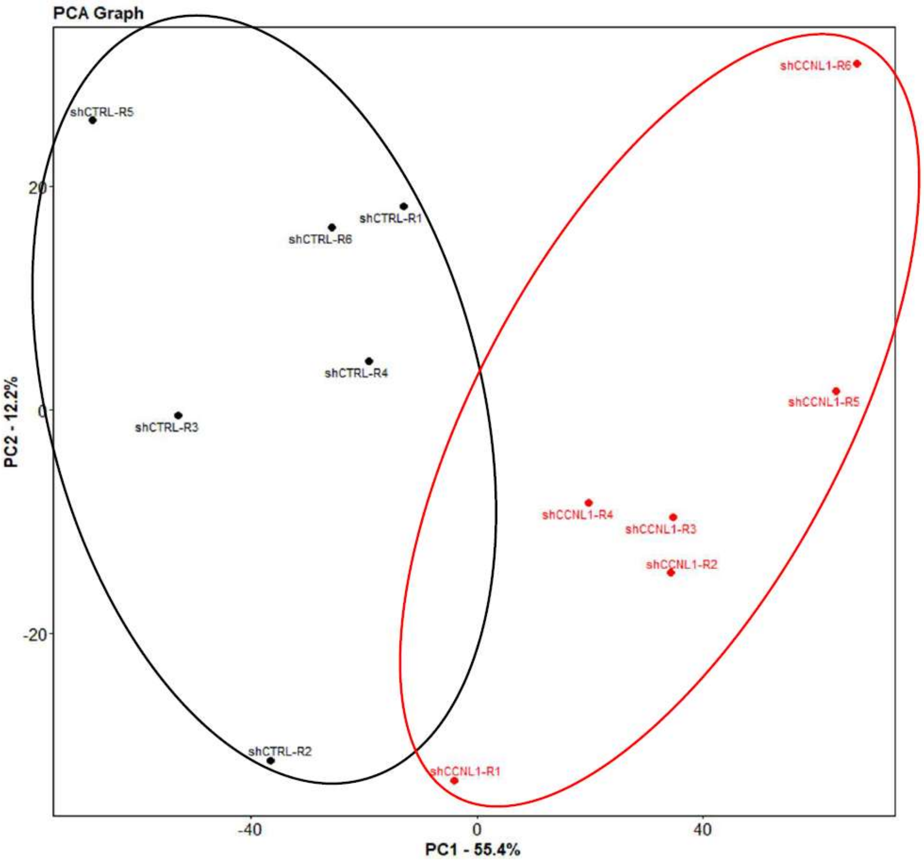

Supplement: Supplementary file 1 [file viruses-18-00545-s001.zip › viruses-4262821-supplementary-figures.pdf]
